# Orthostatic hypotension as a marker of increased arterial stiffness in diabetes mellitus

**DOI:** 10.1007/s00592-026-02704-6

**Published:** 2026-05-06

**Authors:** Merve Oruc, Ozant Helvacı, Ahmet Oruc, Ulver Derici

**Affiliations:** 1 Department of Nephrology, Meram School of Medicine, Necmettin Erbakan University, Konya, Turkey; 2 Department of Nephrology, Gazi University Faculty of Medicine, Ankara, Turkey; 3 Department of Medical Oncology, Meram School of Medicine, Necmettin Erbakan University, Konya, Turkey

**Keywords:** Orthostatic hypotension, Arterial stiffness, Pulse wave velocity, Diabetes mellitus, Cardiovascular autonomic neuropathy

## Abstract

**Background:**

Orthostatic hypotension (OH) is associated with adverse cardiovascular outcomes and may reflect underlying autonomic and vascular dysfunction. Arterial stiffness is a key determinant of cardiovascular risk; however, its relationship with OH in patients with diabetes mellitus (DM) remains unclear.

**Objective:**

To evaluate factors associated with OH and investigate the relationship between arterial stiffness parameters and OH in patients with DM.

**Methods:**

This single-center cross-sectional study included 193 patients with DM. Orthostatic blood pressure was measured in the supine position and 3 min after standing. Arterial stiffness was assessed using oscillometric pulse wave velocity (PWV) and related parameters with the Mobil-O-Graph device. Clinical, laboratory, and medication data were analyzed. Logistic regression analyses were performed to identify factors associated with OH.

**Results:**

OH was present in 56 patients (29%). Patients with OH had significantly higher central blood pressure and arterial stiffness parameters, including PWV, augmentation pressure, and augmentation index. In multivariate analysis, female sex, older age, diabetic neuropathy, and PWV were independently associated with OH. PWV remained significantly associated with OH after adjustment for confounders. No significant differences were observed between groups regarding antihypertensive medication classes.

**Conclusion:**

In patients with DM, OH is independently associated with increased arterial stiffness and diabetic neuropathy. These findings suggest a link between orthostatic blood pressure dysregulation and adverse vascular characteristics. Prospective studies are needed to clarify causal relationships and clinical implications.

## Introduction

Cardiovascular autonomic neuropathy is a frequently overlooked and difficult-to-manage complication in patients with diabetes mellitus. CAN occurs when the autonomic nerves regulating the heart and blood vessels suffer damage, resulting in impaired control of heart rate and vascular dynamics. Several studies have explored the prevalence of CAN among patients with both type 1 and type 2 diabetes, reporting rates ranging from 17% to 66% in type 1 DM and from 31% to 73% in type 2 DM. A late and severe complication of CAN is OH, characterized by dysfunctional hemodynamic blood pressure regulation during the transition from a supine to an upright posture. Orthostatic hypotension is the second most common cause of syncope. Unfortunately, these patients are often misdiagnosed and face a more unfavorable prognosis with a higher risk of death [1, 2].

Assessing arterial stiffness is a crucial method in cardiovascular risk evaluation, with PWV showing an inverse relationship to arterial compliance. Numerous invasive and non-invasive techniques exist for PWV assessment. Among the noninvasive methods, oscillometric Pulse Wave Velocity measurement stands out for its cost-effectiveness, reproducibility, and ease of use, providing an estimation of aortic and proximal vessels’ stiffness [3, 4]. Additional parameters, such as AP, Ai@75 are also employed in determining arterial stiffness.

Arteriosclerosis can be seen in diabetic patients from an early age. With many multifactorial effects such as age, hyperglycemia, smoking, dyslipidemia and obesity, the arterial system loses its elasticity and progressive hardening occurs. Studies have shown that in diabetic patients, the mean aortic blood pressure (MAP), PWV, pulse pressure (PP) and the age, gender and heart rate adjusted increase index are significantly increased [5].

Some studies suggest that arterial stiffness desensitizes baroreceptor reflexes to postural changes. Baroreceptors in the carotid and aorta are located in the walls of elastic arteries and sense blood pressure levels via stretch-sensitive nerve pathways. The loss of the ability to stretch under blood pressure due to arterial stiffness directly impairs baroreceptor function [6]. However, the relationships between the presence of OH and arterial stiffness remain unclear.

In light of these studies, we aimed to evaluate factors associated with the presence of OH in patients with diabetes mellitus. Additionally, we aimed to investigate the relationship between the degree of arterial stiffness and the presence of OH by measuring arterial stiffness parameters in these patients.

## Methods

### Study participants

During the study period, 250 consecutive patients who attended the Internal Medicine, Nephrology, and Endocrinology outpatient clinics of Gazi University Faculty of Medicine were screened for eligibility. 22 patients declined participation. The remaining patients were assessed according to predefined inclusion and exclusion criteria. After exclusions, a total of 193 patients with diabetes mellitus underwent comprehensive clinical and laboratory evaluation and were included in the final analysis.

The inclusion criteria were age ≥ 18 years and a diagnosis of DM. DM was defined as the use of antidiabetic medication, HbA1c ≥ 48 mmol/mol (6.5%), fasting plasma glucose ≥ 126 mg/dL, or a random plasma glucose ≥ 200 mg/dL.

All patients were evaluated in the morning after an overnight fast and before taking their routine medications, including antihypertensive agents, in order to eliminate acute hemodynamic effects and standardize measurements. Patients rested in the supine position for at least 5 min prior to blood pressure assessment. Blood pressure was measured in the supine position and repeated 3 min after standing using the same calibrated automatic device. Orthostatic hypotension was defined according to guideline-based criteria as a decrease of ≥ 20 mmHg in systolic blood pressure and/or ≥ 10 mmHg in diastolic blood pressure within 3 min of standing. We focused on classical orthostatic hypotension and therefore based our assessment on the 3-minute measurement, which is widely accepted in international consensus definitions [7] .Orthostatic symptoms (dizziness, lightheadedness, blurred vision, or history of syncope) were systematically assessed through structured questioning.

Diabetic neuropathy was defined based on the presence of distal symmetric symptoms such as burning sensation, numbness, tingling, and sensory disturbances in the hands and feet. Neuropathy assessment was performed through standardized systematic questioning. Objective diagnostic tests such as monofilament testing, vibration perception threshold, or nerve conduction studies were not routinely performed due to the cross-sectional outpatient design of the study.

Information regarding antihypertensive medication use, including ACE inhibitors/ARBs, beta-blockers, calcium channel blockers, diuretics, and alpha-blockers, was recorded and included in the statistical evaluation.

Body mass index (BMI) was calculated as weight divided by height squared (kg/m²), and patients with a BMI > 25 kg/m² were classified as overweight. Estimated glomerular filtration rate (eGFR) was calculated using the CKD-EPI formula.Exclusion criteria included age < 18 years, eGFR < 60 mL/min/1.73 m², pregnancy, active infection, active bleeding, central nervous system–related autonomic dysfunction, and psychiatric disorders.

Patients with eGFR < 60 mL/min/1.73 m² were excluded because chronic kidney disease has an independent effect on both arterial stiffness and orthostatic hypotension. This exclusion was intended to minimize potential confounding and to obtain a more homogeneous diabetic population. Additionally, by focusing on patients without advanced renal impairment, we aimed to identify orthostatic hypotension at an earlier stage of the disease process, thereby enabling earlier risk stratification and timely intervention.

### Study design

This study was designed as a single-center, non-interventional, cross-sectional descriptive investigation. Patients enrolled in the study underwent individual evaluations within outpatient clinic settings. Recorded data included demographic characteristics, type and duration of diabetes mellitus, diagnosed DM complications, Chronic Kidney Disease (CKD) stages, symptoms of orthostatic hypotension and concurrent laboratory test results.

### Postural blood pressure measurement

Arterial blood pressure measurements were performed in accordance with the diagnostic criteria for orthostatic hypotension. It was ensured that the patients had not smoked, consumed caffeinated beverages or eaten within the last 30 min. An Omron-M3 Hem7200 (Omron Healthcare Co, Ltd, Kyoto, Japan) device was connected to the left upper arm and arterial blood pressure was measured in the supine position and 3 min after standing up.

### Arterial stiffness measurement

**Fig. 1:**
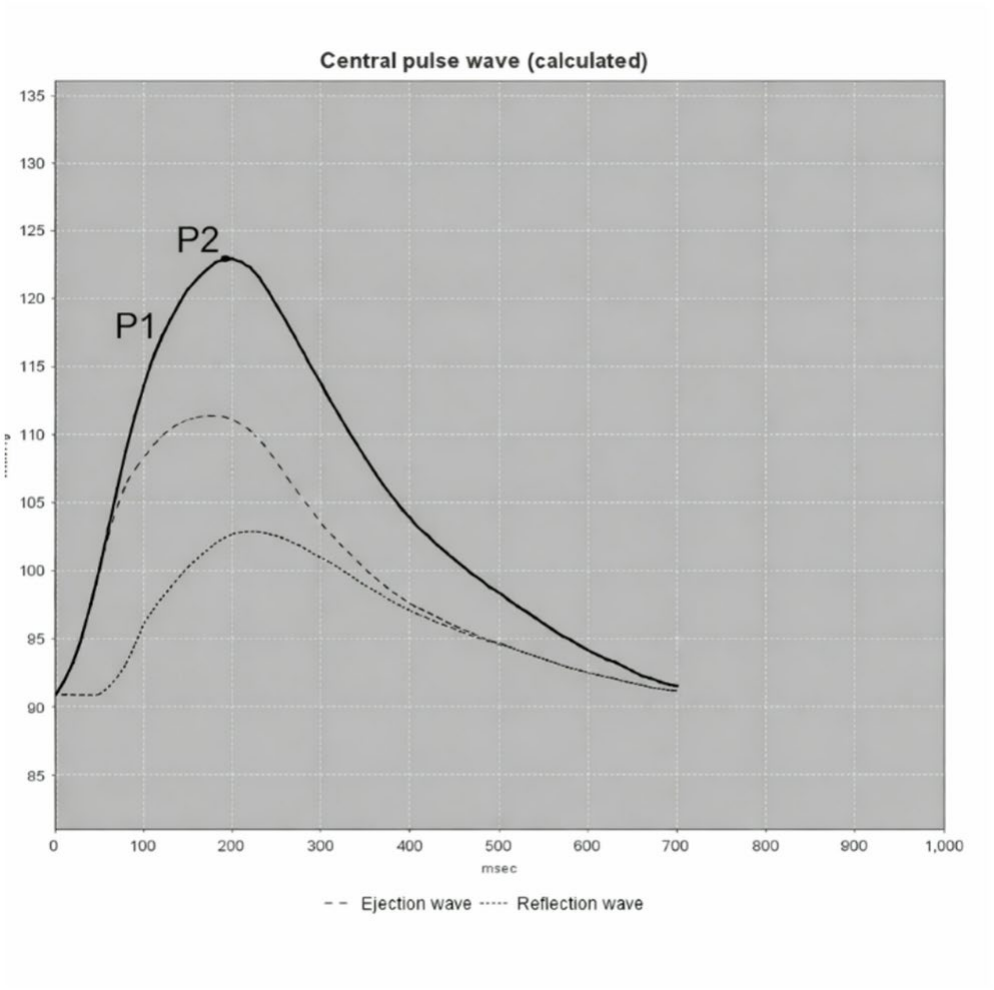
Calculated Central Pulse Waveform Showing P1 and P2 Peaks

The Mobil-O-Graph 24 h-PWA monitor (I.E.M. GmbH, Stolberg, Germany) was employed for arterial stiffness (AS) measurement, concurrent with OH measurements. All arterial stiffness measurements were performed in the morning after at least 5 min of rest in a quiet room under standardized conditions. The Mobil-O-Graph device was programmed to obtain three consecutive measurements automatically, and the mean values were used for statistical analysis.During cuff decompression, early and late systolic waves, along with the diastolic wave, generated a pressure wave at the occlusion site. These waves were transmitted to the cuff via the upper arm and generalized. The monitor’s pressure sensors detected pressure changes, amplified them using a specialized tonometer, and finalized the measurements. PWV was calculated using the Korteweg-Moens equation: PWV=√(E.h/2rρ), where E: represents the wall elasticity coefficient, h: is the vessel wall thickness, r: denotes the vessel radius, and ρ: stands for blood density [8]. In the presence of arterial stiffness, PWV increases, causing the reflected wave to return earlier, contributing to the forward wave and elevating central blood pressure. After systole, the pressure at peak flow (P1) forms a notch in the pressure wave until the reflected wave is added, and the pressure resulting from this addition is termed P2 (Fig. 1). The difference between P2 and P1 is identified as AP, and the ratio of AP to NB is equivalent to the augmentation index [9].

### Statistical analysis

The data were analyzed using SPSS version 22.0. The assumption of normality for continuous variables was assessed using the Kolmogorov-Smirnov test. In the descriptive statistics section, categorical variables were presented as numbers and percentages, normally distributed numerical variables as mean ± standard deviation, and non-normally distributed numerical variables as median values. The normal distribution of continuous variables was assessed through visual and analytical tests. The Mann-Whitney U test was employed for comparing two groups when data did not adhere to normal distribution, and the Kruskal-Wallis Test was applied for comparisons involving three groups or more with non-normally distributed data. Chi-square tests were used for comparing categorical variables between independent groups. Factors influencing OH were first tested using univariate logistic regression analysis. Variables with a p-value < 0.05 were included in multivariate logistic regression analysis. Multicollinearity among arterial stiffness parameters (PWV, augmentation index, augmentation pressure, central pulse pressure, and central systolic blood pressure) was assessed using variance inflation factor (VIF) analysis. Variables demonstrating significant collinearity were not entered simultaneously into the multivariate model. Age was included in the multivariate model as a clinically relevant confounder regardless of its univariate significance.Receiver Operating Characteristic (ROC) analysis was performed for the PWV cutoff point. The area under the curve was 0.612 (*p* = 0.043), and the cutoff point was 8.25 m/s. In this study, a statistical significance level of p˂0.05 was considered.

## Results

A total of 193 patients with DM were included in the evaluation. Systolic blood pressures (SBP) in the supine and upright positions were 136.5 ± 18.11 mmHg and 127.69 ± 19.23 mmHg, respectively. Diastolic blood pressures (DBP) in the supine and upright positions were 81.71 ± 9.21 mmHg and 84.18 ± 10.78 mmHg, respectively. Out of the total, 56 patients were diagnosed with orthostatic hypotension.

**Fig. 2:**
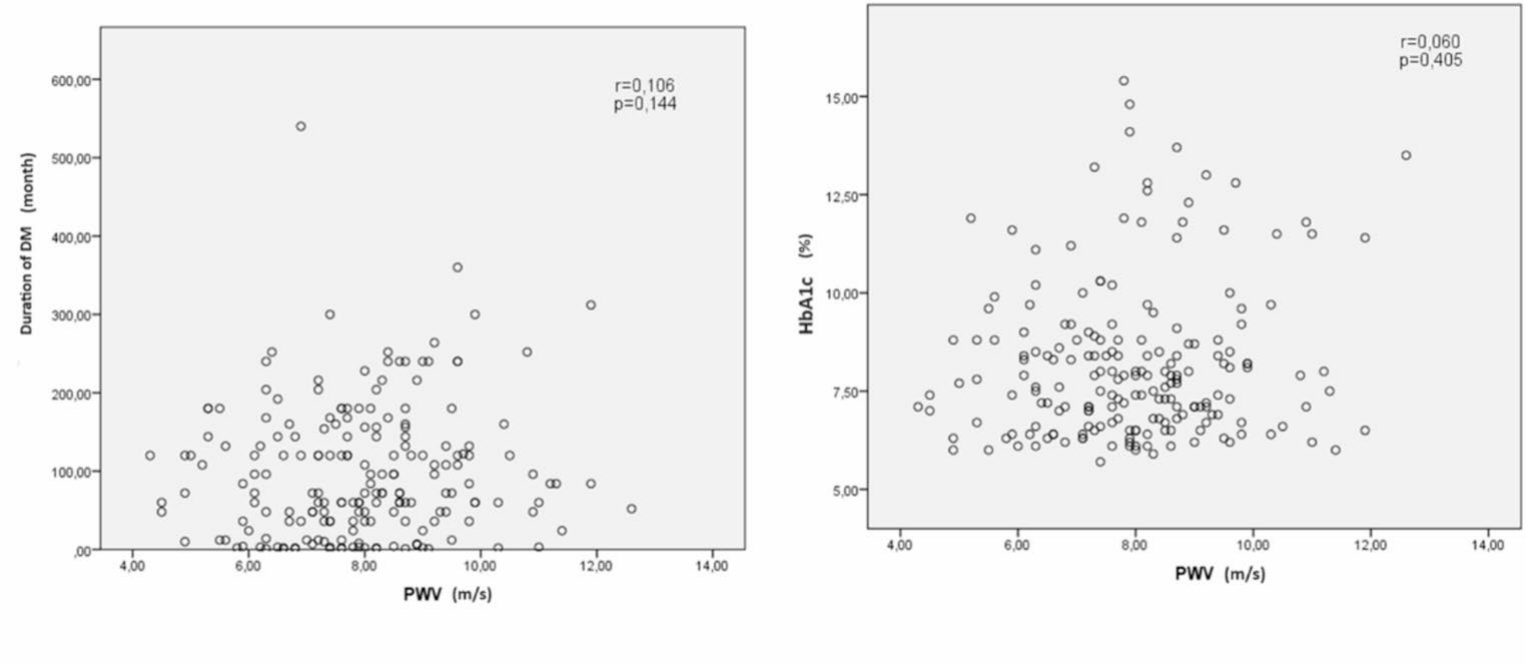
Correlation Between Duration Of Diabetes, Hba1c and PWV. PWV: Pulse wave velocity

Out of the total patients, 95 were female (49.2%), and 98 were male (50.8%), with a mean age of 52.78 ± 12.24 years. A significant number of patients, 141, had comorbidities other than DM. Among the 56 patients diagnosed with OH, 37 were female and 19 were male, showing a statistically significant difference (*p* = 0.03). In the assessment of arterial stiffness parameters between genders, central and peripheral pulse pressures were 46 (21–99) mmHg and 47 (22–108) mmHg, respectively, while augmentation pressure and augmentation index were 11 (2–35) mmHg and 31% (4–52), respectively, in female patients, and these values were higher than those in male patients. (*p* = 0.02, *p* = 0.03, *p* < 0.001, *p* < 0.001, respectively). No significant differences were observed between the groups in terms of BMI, smoking, and alcohol consumption. Patient age did not significantly differ between the OH and non-OH groups; however, SBP, peripheral pulse pressure (pPP), augmentation pressure (AP), and PWV values increased with increasing patient age. Among patients with OH, 37 (49.3%) reported experiencing OH symptoms, indicating a significant difference between the groups (*p* < 0.001). There were no significant differences between the groups in terms of diabetes type (type 1 vs. type 2) or laboratory parameters. (Table 1). Additionally, no significant correlations were found between the duration of DM and HbA1c levels and PWV (Fig. 2). OH was identified in 20 (52.6%) out of 38 patients with diabetic neuropathy, signifying a notable difference between the groups.

**Table 1:** Evaluation of patient groups according to clinical and laboratory characteristics

Gender (male/female)	Patients with orthostatic hypotension (*n*:56)	Patients without orthostatic hypotension (*n*:137)	*p* value
	37 (66.1%) /19 (33.9%)	58 (42.3%) /79 (57.7%)	0.03^a^
Age Med (Min-Max)	53 (21-72)	53 (19-79)	0.85^&^
BMI Med (Min-Max)	29.35 (18.75-50.15)	28.7 (19.6-59.5)	0.19^&^
Normal BMI	9 (16.1%)	32 (23.4%)	0.35^a^
Overweight BMI	47 (83.9%)	105 (76.6%)	
Never smoked	36 (64.3%)	79 (57.7%)	0.49^a^
Active Smoker / Exsmoker	20 (35.7%)	58 (42.3%)	
Alcohol use/non-use	3 (5.4%)/ 53 (94.5%)	16 (11.7%)/ 121 (88.3%)	0.28^a^
Comorbidity Present/ Absent	38 (67.9%) / 18 (32.1%)	93 (67.9%) / 44 (32.1%)	0.99^a^
OH symptom experience n(%)	37 (49.3%)	38 (50.7%)	**<0.001** ^**a**^
Type 2DM n (%)	55 (30.4%)	126 (69.6%)	0.185^a^
Type 1 DM n (%)	1 (8.3%)	11 (91.7%)	0.193^a^
Diabetic nephropathy (%)	17 (28.3%)	43 (71.7%)	0.888^a^
Diabetic retinopathy (%)	8 (32.0%)	17 (68.0%)	0.814^a^
Diabetic neuropathy (%)	20 (52.6%)	18 (47.4%)	**0.010** ^a^
Duration of DM Med (Min-Max)	78 (1-360)	72 (1-540)	0.785^b^
HbA1c, %	7.9 (5.9-14.8)	7.7 (5.7-15.4)	0.529^b^
FSP, mg/dl	146 (76-371)	141 (76-462)	0.684^b^
BUN, mg/dl	13.5 (7-29)	14 (4-87)	0.732^b^
GFR, mL/dak/1.73 m^2^	106.2 (63-228)	105.7 (60-186)	0.774^b^
Cre, mg/dl	0.69 (0.48-1.16)	0.78 (0.44-1.16)	0.106^b^
Spot urine protein, mg/dl	105 (14.4-2255)	96.5 (4-1060)	0.140^b^
Spot urine creatine, mg/dl	93.7 (18.1-280)	106 (7-479)	0.331^b^
Spot urine albumine, mg/dl	0.94 (0.02-138.4)	1.05 (0.05-116.5)	0.984^b^

^a^chi-Square Test

^b^Mann-Whitney U Test

*DM* Diabetes Mellitus, *BMI* Body Mass Index, *OH* Orthostatic Hypotension, *Ccbs* Calcium Channel Blockers, *ACE* Angiotensin-Converting Enzyme, *Arbs* Angiotensin II Receptor Blockers, *Mras* Mineralocorticoid Receptor Antagonists, *FSP* Fasting Plasma Glucose, *Cre* Creatine, *BUN* Blood Urine Nitrogen, *GFR* Glomerular Filtration Rate, *Med* Median, *Min* Minimum, *Max* Maximum. Note: Statistically Significant Values Are Shown in Bold (*p* < 0.05)

There were no significant differences between the OH and non-OH groups regarding the use of antihypertensive medication classes, including calcium channel blockers, beta-blockers, ACE inhibitors, ARBs, diuretics (loop and thiazide), mineralocorticoid receptor antagonists, and alpha-blockers (all *p* > 0.05).

**Fig. 3:**
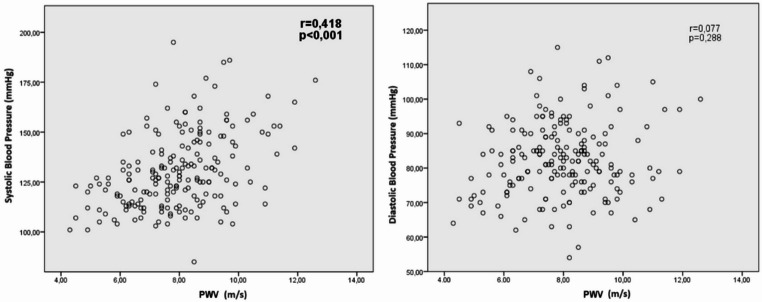
Correlation Between SBP, DBP And PWV. PWV: Pulse wave velocity

Peripheral and central SBP, pulse pressure and mean arterial blood pressure were significantly higher in the OH group than in the group without OH. PWV and other arterial stiffness parameters were significantly higher in the OH group (Table 2). A positive correlation was observed between systolic blood pressure and PWV, while no significant correlation was found between diastolic blood pressure and PWV (Fig. 3).

**Table 2:** Evaluation of arterial stiffness measurement parameters after monitoring in patient groups

		Patients with orthostatic hypotension (*n*:56)	Patients without orthostatic hypotension (*n*:137)	*P* value^b^
SBP, mmHg	Med (Min-Max)	140.5 (104–186)	125 (85–195)	< 0.001
DBP, mmHg	Med (Min-Max)	81 (54–112)	82 (57–115)	0.252
pPP, mmHg	Med (Min-Max)	52.5 (31–108)	42 (22–91)	**< 0.001**
MAP, mmHg	Med (Min-Max)	102 (81.3-136.3)	97.3 (66.3-141.6)	**0.008**
Pulse rate, bpm	Med (Min-Max)	84 (55–117)	81 (39–111)	0.081
cCBP, mmHg	Med (Min-Max)	138 (102–188)	125 (83–178)	**0.001**
cDBP, mmHg	Med (Min-Max)	82 (56–112)	83 (57–118)	0.465
cPP, mmHg	Med (Min-Max)	50 (27–99)	42 (21–87)	**0.001**
TVR, mmHg/ml/min	Med (Min-Max)	1.2 (0.8–1.6)	1.2 (0.8–1.7)	0.070

^b^Mann-Whitney U test, *CBP* Systolic Blood Pressure, *DBP* Diastolic Blood Pressure, *pPP* Peripheral Pulse Pressure, *MAP* Mean Arterial Pressure, *cDBP* Central Diastolic Blood Pressure, *cPP* Central Pulse Pressure, *TVR* Total Vascular Resistance

In the univariate logistic regression analysis, age, sex, presence of diabetic neuropathy, cCBP, cPP, TVR, Ai@75, PWV and spot urine protein were included as potential predictors of orthostatic hypotension. Due to multicollinearity, AP was excluded from the analysis because of its high correlation with cPP. The remaining variables showed variance inflation factor (VIF) values < 5, indicating no significant multicollinearity. Variables that were statistically significant in the univariate analysis were subsequently entered into the multivariate logistic regression model. In the multivariate logistic regression analysis, female sex, older age, diabetic neuropathy, and PWV were independently associated with orthostatic hypotension (Table 3).

**Table 3:** Logistic Regression Analysis of Factors Affecting Orthostatic Hypotension

Gender	Univariate Logistic regression analysis Crude OR (95% CI; *p*)	Multivariate Logistic regression analysis Adjusted OR (95% CI; *p*)
Male	**Ref**	**Ref**
Female	**2.65 (1.38–5.07; p = 0.009)**	**3.38 (1.47–7.74; p = 0.004)**
Age < 53	Ref	**Ref**
Age ≥ 53	1.11 (0.59–2.09; *p* = 0.723)	**4.2 (1.57–11.55; p = 0.004)**
Presence of diabetic neuropathy		
Without Diabetic Neuropathy	**Ref**	**Ref**
Diabetic Neuropathy	**2.77 (1.32–5.77; p = 0.007)**	**3.52 (1.36–9.15; p = 0.010)**
*cCBP*,* mmHg*		
cCBP < 131mmHg	**Ref**	**Ref**
cCBP ≥ 131mmHg	**2.49 (1.32–4.71; p = 0.005)**	**3.02 (1.60–8.58; p = 0.038)**
*cPP*,* mmHg*		
cPP < 47 mmHg	**Ref**	**Ref**
cPP ≥ 47 mmHg	**2.83 (1.49–5.38; p = 0.001)**	**2.07 (0.67–6.39; p = 0.206)**
*TVR*,* mmHg/ml/min*		
TVR < 1.20 mmHg/ml/min	Ref	
TVR ≥ 1.20 mmHg/ml/min	1.40 (0.73–2.69; *p* = 0.308)	
*AP*,* mmHg*		
AP < 10.5 mmHg	**Ref**	
AP ≥ 10.5 mmHg	**2.39 (1.27–4.51; p = 0.007)**	
*AI@75%*		
AI@75 < 30%	**Ref**	**Ref**
AI@75 ≥ 30%	**3.40 (1.78–6.51; p < 0.001**)	**1.59 (0.70–3.61; p = 0.264**)
*PWV m/s*		
PWV ≤ 8.25 m/s	**Ref**	**Ref**
PWV > 8.25 m/s	**8.41 (4.11–17.19; p < 0.001)**	**18.7 (6.20-56.48; p < 0.001)**
*Spot urine protein*		
Spot urine protein < 102 mg/dl	Ref	
Spot urine protein ≥102 mg/dl	1.58 (0.84–2.98; *p* = 0.151)	

*OR* odds ratio, *CI* Confidence Interval, *Ref* Reference, *CBP* Systolic Blood Pressure, *cPP* Central Pulse Pressure, *TVR* Total Vascular Resistance, *AP* Augmentation Pressure, *AI@75* Augmentation Index, *PWV* Pulse Wave Velocity

## Discussion

OH, although commonly associated with congestive heart failure, neurodegenerative diseases, and elderly individuals, is also prevalent in patients with diabetes and hypertension. In this study, we investigated factors independently associated with OH and its relationship with arterial stiffness parameters in a diabetic population.

Orthostatic intolerance is more common in women than men and the mechanism of this gender difference has been explored through several hypotheses. Female sex was independently associated with OH in our cohort, consistent with previous reports suggesting sex-related differences in autonomic regulation and vascular responses. Although the precise mechanisms could not be evaluated, hormonal and physiological differences may partially explain this association [10–12].

Autonomic nervous system and baroreceptor sensitivity tend to decrease with age, and the prevalence of OH is expected to increase accordingly. OH increases the risk of coronary heart disease and all-cause mortality in the elderly. Atherosclerosis and increased arterial stiffness in old age are estimated to be contributing factors to this phenomenon [10–12]. Although orthostatic hypotension is traditionally considered more prevalent in older populations, the relatively young mean age of our cohort may reflect the contribution of diabetic autonomic dysfunction, which can occur independently of chronological aging. In patients with diabetes mellitus, subclinical cardiovascular autonomic impairment may develop earlier and contribute to impaired postural blood pressure regulation. Therefore, the observed prevalence of OH (29%) in our study may indicate underlying autonomic and vascular dysfunction rather than age-related hemodynamic changes alone. Additionally, since no significant differences were observed in antihypertensive medication classes between groups, the association between OH and arterial stiffness parameters is unlikely to be explained solely by treatment-related effects. These findings suggest that vascular and autonomic factors may play a central role in the development of OH in diabetic patients, even in relatively younger individuals [13]. Notably, in multivariate logistic regression analysis, older age emerged as an independent factor associated with orthostatic hypotension, suggesting that age may contribute to OH when adjusted for other clinical and vascular variables. However, systolic blood pressure, pulse pressure, and arterial stiffness parameters increased with age, consistent with the well-established relationship between vascular aging and hemodynamic changes.

Antihypertensive medications are recognized contributors to orthostatic blood pressure changes and therefore represent a potential confounding factor in studies evaluating orthostatic hypotension [14]. In the present study, however, the distribution of antihypertensive drug classes did not differ significantly between the OH and non-OH groups. This finding suggests that the observed associations between OH and arterial stiffness parameters are unlikely to be solely explained by differences in antihypertensive treatment patterns. Nevertheless, the potential influence of chronic pharmacodynamic effects cannot be entirely excluded.

OH is often asymptomatic or presents with few specific symptoms, particularly in the early stages. The risk of falls increases as the severity of symptoms worsens during positional changes, and symptomatic OH may adversely affect daily functioning and quality of life [15]. Previous studies have shown that only a small percentage of patients with OH experience noticeable symptoms [16–18]. The difficulty of performing CAN diagnostic tests, particularly in asymptomatic outpatients, is well known. In our study, structured questioning revealed a higher frequency of neuropathic symptoms among patients with OH, supporting the association between orthostatic blood pressure abnormalities and diabetic neuropathic involvement. These findings emphasize the importance of systematic symptom assessment in diabetic patients.

A significant association was found between OH and diabetic neuropathy and diabetic neuropathy was found to be associated with orthostatic hypotension; however, this finding should be interpreted cautiously given the symptom-based definition without objective confirmation. However, the absence of standardized autonomic function testing limits definitive conclusions regarding the presence or severity of CAN in this cohort. Nevertheless, the observed association between neuropathy and orthostatic hypotension is pathophysiologically plausible and consistent with the known spectrum of diabetic autonomic dysfunction.

There is a close association between OH and retinopathy and nephropathy, microvascular complications of diabetes. The presence of orthostatic hypotension is strongly associated with a higher prevalence of microvascular complications in diabetic patients [11]. In our cohort, no significant association was observed between OH and nephropathy or retinopathy; however, this finding should be interpreted cautiously due to the exclusion of patients with reduced renal function.

The baroreflex mechanism plays a central role in maintaining blood pressure stability during postural changes. Impairment in baroreflex sensitivity has been associated with autonomic dysfunction in patients with diabetes and carries prognostic significance [19–22]. In the present study, patients with OH exhibited higher arterial stiffness parameters, including PWV, augmentation pressure, and augmentation index. Notably, PWV remained independently associated with OH in the multivariable analysis, with an odds ratio of 18.7. However, the relatively high odds ratio observed for PWV (OR = 18.7), together with the limited number of outcome events (*n* = 56), raises concerns regarding the stability of the multivariate model. This may indicate potential overestimation of the effect size and reduced robustness of the findings. Therefore, these findings should be interpreted with caution and confirmed in larger, adequately powered prospective cohorts. Collectively, these results support the hypothesis that increased arterial stiffness may contribute to impaired postural blood pressure regulation, possibly through reduced baroreceptor sensitivity and diminished autonomic compensation.

## Limitations

This study has several limitations. First, although patients were recruited consecutively, the single-center design may limit the generalizability of the findings. Second, by excluding patients with eGFR < 60 mL/min/1.73 m², we intentionally focused on earlier stages of diabetic vascular involvement; however, the results may not be applicable to individuals with advanced chronic kidney disease.Diabetic neuropathy was defined based on symptom assessment without objective confirmatory testing, which may have introduced misclassification bias and led to potential under- or overestimation of neuropathy prevalence.Although the Mobil-O-Graph device has been validated for oscillometric estimation of PWV, it does not represent the gold-standard carotid–femoral PWV measurement. Therefore, arterial stiffness parameters should be interpreted within the methodological constraints of oscillometric brachial-derived estimations. In addition, standard autonomic function tests (such as heart rate variability analysis, Valsalva maneuver, or deep breathing tests) were not performed; therefore, orthostatic hypotension could not be definitively attributed to advanced cardiovascular autonomic neuropathy.The relatively small number of patients with orthostatic hypotension (*n* = 56) represents a limitation of this study, particularly for multivariate modeling. Although the number of variables included in the regression analysis was carefully limited, the modest event count may have affected the precision of the estimates and increases the potential risk of model instability. In addition, the relatively high effect size observed for PWV in the multivariate model should be interpreted with caution, as it may reflect model instability related to the limited number of events. Although PWV was dichotomized based on ROC-derived cutoff values for clinical interpretability, this approach may have resulted in loss of statistical power and potential residual confounding. Continuous modeling of PWV might have provided a more precise estimation of its relationship with OH.

## Conclusion

In this study, diabetic patients with orthostatic hypotension exhibited higher arterial stiffness parameters compared to those without OH. Female sex, older age, diabetic neuropathy, higher central blood pressure, and increased PWV were independently associated with the presence of OH. These findings suggest a potential association between orthostatic blood pressure dysregulation and adverse vascular characteristics in patients with diabetes.

While routine management of diabetes already includes cardiovascular risk assessment, the identification of increased arterial stiffness in patients with orthostatic hypotension may contribute to improved cardiovascular risk stratification when interpreted alongside conventional risk markers.

Although causal relationships cannot be established due to the cross-sectional design, assessment of arterial stiffness parameters in patients with OH may provide additional insight into cardiovascular risk. Further prospective studies are warranted to clarify temporal and mechanistic relationships.
